# Coupling between γ-irradiation and synchrotron-radiation-based XAFS techniques for studying Mn-doped ZnO nanoparticles

**DOI:** 10.1107/S1600577522006439

**Published:** 2022-07-15

**Authors:** N. G. Imam, Messaoud Harfouche, A. A. Azab, S. Solyman

**Affiliations:** a Experimental Nuclear Physics Department (Solid State Laboratory), Nuclear Research Center (NRC), Egyptian Atomic Energy Authority (EAEA), Cairo 13759, Egypt; b Synchrotron-Light for Experimental and Scientific Applications in the Middle East (SESAME), PO Box 7, Allan 19252, Jordan; c Solid State Physics Department, Physics Research Institute, National Research Centre, 33 El Bohouth Street, Dokki, 12622 Giza, Egypt; d Physics Department, Faculty of Science, Zagazig University, Zagazig, Egypt; University of Essex, United Kingdom

**Keywords:** synchrotron radiation, γ-ray irradiation, XAFS, XANES, pre-edge fitting, LCF, EXAFS, XRD, Rietveld refinement, magnetism, Mn-doped ZnO, SESAME

## Abstract

γ-Irradiation and synchrotron-radiation-based X-ray absorption fine-structure (XAFS) spectroscopy have been used to induce structure disorder through the interaction of γ-rays (200 kGy) with fabricated Mn-doped ZnO nanoparticles (NPs) and then to examine thoroughly the resultant structural change. The extracted electronic/fine XAFS structural parameters reflect a compositional and γ-irradiation co-dependence. The average crystal structure of samples prepared by the sol-gel method was investigated by X-ray diffraction (XRD). A detailed structural XRD data analysis was carried out by applying a Rietveld refinement using the *MAUD* program. XAFS spectra were collected at the Zn *K*-edge (9659 eV) in transmission mode and at the Mn *K*-edge (6539 eV) in fluorescence mode. Direct evidence of the solubility of Mn ions in the ZnO structure was demonstrated by fitting the extended-XAFS (EXAFS) signal. Near-edge XAFS (XANES) analysis provided the oxidation states of Zn and Mn ions through fingerprint XANES spectra of the sample along with those of standard compounds. Linear combination fitting showed that the most fit chemical forms of Zn and Mn in the samples are ZnO and MnO, respectively. The oxidation states of both Zn and Mn XAFS absorbers were confirmed from pre-edge fitting. The results of the magnetic measurements were explained in light of the average and electronic/local structural information obtained from XRD, XANES and EXAFS techniques. The magnetic properties of the samples translate into an induced change in the average crystal and electronic/local structures upon Mn concentration change and γ-irradiation. XRD confirmed the successful preparation of hexagonal Mn-doped ZnO NPs with a crystallite size in the range 33–41 nm. Both XRD and EXAFS analysis detected a minor amount of Mn_3_O_4_ as a secondary phase. XANES and EXAFS provided information exploring the outstanding potential of the utilized protocol for detecting precisely the presence of the secondary phase of Mn_3_O_4_, which changes with Mn content (*x*). Mean-square relative displacement (σ^2^) values extracted from the EXAFS fitting were found to grow for Zn–Zn/Mn paths demonstrating the substitution of Mn/Zn into Zn crystal sites. The EXAFS analysis explains the reasons behind the enhancement in the magnetic properties and shows that the Mn doping content at *x* = 0.05 produces the most local atomic disorder in ZnO NPs. There is a strong harmony among the XRD, XANES, EXAFS and magnetization behavior of the Mn-doped ZnO NPs. Maximum magnetization was acquired at an Mn content of 0.05. γ-Ray-irradiated Zn_1–*x*_Mn_*x*_O NPs are recommended as optimized candidates for showing the diversity of the applications.

## Abstract 2

X-ray absorption fine-structure (XAFS) spectroscopy carried out at the SESAME synchrotron has provided detailed fine (local/electronic) structural information on pristine and γ-irradiated Mn–ZnO nanoparticles. XAFS describes structural changes upon γ-irradiation and Mn-doping by determining very small amounts of the secondary phase of Mn_3_O_4_ along with structure distortion, and their impact on improving the magnetic properties of ZnO:Mn.

## 1. Introduction

Several wide-band-gap semiconductors such as ZnO, GaAs, InAs and GaN can demonstrate ferromagnetism above room temperature when doped with diluted transition metals like Mn, as in our case study in this work. Diluted magnetic semiconductors (DMSs) are attracting attention as promising spintronic semiconductors, with potential applications in semiconductor devices (Moontragoon *et al.*, 2013 ▸). Ferromagnetic materials, at room temperature, use the spin of an electron (spintronic) instead of the charge for reading, writing data and transmitting information for integration with conventional semiconductor technologies (Moontragoon *et al.*, 2013 ▸; Singhal *et al.*, 2009 ▸). Recently, diluted magnetic oxides such as ZnO, TiO_2_ and CeO_2_ doped with various transition metals such as Mn, Co, Fe and Ni have been widely investigated theoretically and experimentally in order to predict their magnetic properties (Min *et al.*, 2004 ▸; Pan *et al.*, 2008 ▸; Tiwari *et al.*, 2017 ▸; Ozkendir *et al.*, 2016 ▸; Viswanatha *et al.*, 2004 ▸). Mn-doped ZnO, as a DMS material, is the most popular candidate among DMS oxides because it is easier to produce than other oxide materials and is widely used in optoelectronic devices due to its own impressive optical properties. The substantial objective of fabricating these types of DMSs, which could be in bulk samples or thin films or on the nanoscale, is to realize intrinsic ferromagnetism inside the material matrix (Labuayai *et al.*, 2009 ▸; Prabhakar *et al.*, 2012 ▸; Krohns *et al.*, 2011 ▸). Generally, ZnO NPs have a relatively high excitation binding energy (60 MeV) and a large band gap (3.3 eV) corresponding to the specific particle size (at room temperature). As mentioned above, due to the unique optoelectronic performance of ZnO nanostructures, they have wide application in LEDs, lasers, solar cells and optical/radiation sensors (Özgür *et al.*, 2005 ▸; Schmidt-Mende & MacManus-Driscoll, 2007 ▸). Many researchers in nanotechnology have used a variety of methods to synthesize zinc oxide NPs doped with mono-doping or co-doping transition metal ions (Mn, Fe, Co, Ni, Cr); for example, sol-gel (Khorsand Zak *et al.*, 2012 ▸; Liu *et al.*, 2010 ▸), spray (Shinde *et al.*, 2006 ▸), radio-frequency magnetron sputtering (Ashida *et al.*, 2006 ▸), pulsed laser deposition (Tiwari *et al.*, 2002 ▸), metal organic deposition (Tuan *et al.*, 2004 ▸) and co-precipitation (Harsono *et al.*, 2017 ▸; Tan *et al.*, 2014 ▸; Bagheri *et al.*, 2013 ▸). Also, γ-ray irradiation has been used for the preparation of metal oxides (Imam *et al.*, 2021*a* ▸). It is reported that zinc oxide nanocrystals show a weak ferromagnetic nature originating from intrinsic crystalline oxygen and zinc crystalline defects (Morales-Pérez *et al.*, 2021 ▸). Therefore, the magnetic properties of zinc oxide could be enhanced by creating more structure defects and by doping with transition-metal magnetic cations. In our case study, we tailor ZnO NPs to be a more functional material by using γ-ray irradiation to create/induce more structural defects/disorder, as well as doping ZnO NPs with transition elements (Mn) in the concentration range 0.03 ≤ *x* ≤ 0.07.

In the present work, the synchrotron-radiation-based XAFS (SR-XAFS) technique was used at SESAME (Synchrotron-light for Experimental and Scientific Applications in the Middle East, Jordan) to map the fine/local and electronic structural changes in Zn_1–*x*_Mn_*x*_O nanocrystals induced by γ-ray irradiation and/or compositional change effects upon Mn doping. The fine structural change is indirectly monitored and confirmed through observed variations in the magnetic properties. As such, synchrotron-radiation-based XAFS, combining both X-ray absorption near-edge structure (XANES) and extended X-ray absorption fine-structure (EXAFS), is an impressive spectroscopic technique for determining the electronic and local/fine structure surrounding a selected absorbing ion such as the coordination geometry and coordination number, structure disorder, bond length and the oxidation states of the selective target elements (absorbers) (Tan *et al.*, 2014 ▸). Therefore, XAFS is an element-selective technique having the advantage of the ability to tune the energy of the incident photons to the value of either the *K*-edge or the *L*-edge energy of the selected atom (Imam *et al.*, 2021*b* ▸). The necessity of the XAFS technique arises from monitoring the induced changes in the electronic and fine/local structures around absorbing ions in the short-range order of a few angstroms (∼5 Å). This structural change is difficult to monitor from direct X-ray diffraction (XRD) measurements, which determines the average crystal structure in the long-range order (Imam *et al.*, 2019 ▸). Moreover, the XRD technique could not distinguish between different elements with similar scattering factors.

The main aim of this work is to shed light on the coupling between the average crystal and electronic/local fine structural ordering and the magnetic behavior of Mn-doped ZnO NPs; and on the coupling between the γ-irradiation to induce crystal imperfections and synchrotron radiation XAFS (XANES and EXAFS) for mapping the effect of γ-irradiation on the atomic/electronic and local structures in Mn-doped ZnO NPs.

## 2. Experimental procedure

### 2.1. Synthesis procedure and instrumentation

The sol-gel method was used for the synthesis of undoped and Mn-doped zinc oxide nanocrystals according to the formula Zn_1–*x*_Mn_*x*_O (*x* = 0.0, 0.03, 0.05 and 0.07). Briefly, zinc nitrate [Zn(NO_3_)_2_.6H2O] and manganese chloride (MnCl_2_) powders (99.9%) and ethyl­ene glycol were used as precursors. For sample *x* = 0.0, 25 ml of ethyl­ene glycol was stirred at 80°C for 10 min and 0.032 mol of zinc nitrate was added and stirred for 2 h; stirring was then stopped and the temperature was raised to 200°C. The solution was then dried to obtain a xerogel. The collected powders were ground and sintered at 550°C for 3 h under atmospheric air conditions. The same procedure was repeated for the other samples according to the stoichiometric ratios for zinc nitrate and manganese chloride. The obtained prepared powder samples were preliminary characterized using an X-ray diffractometer (ProkerD8-USA) with Cu *K*α radiation (1.54056 Å) over a wide range of Bragg angle (from 20° to 80°). To correct for XRD instrumental broadening, an XRD pattern of LaB_6_ standard sample was collected (Imam *et al.*, 2019 ▸). The magnetic properties were measured using a vibrating sample magnetometer (VSM) (Model 7410, Lakeshore, USA).

### 2.2. XAFS measurements and fitting analysis

In this study, room-temperature Zn and Mn *K*-edge SR-XAFS spectra were acquired at the XAFS/XRF beamline of the SESAME synchrotron facility. SESAME – Synchrotron-Light Source for Experimental Science and Applications in the Middle East – operates at 2.5 GeV with a maximum injection current of 250 mA (Harfouche *et al.*, 2018 ▸). The SESAME XAFS/XRF beamline has been constructed to provide electromagnetic radiation in the hard X-ray region (4700–30000 eV) and is dedicated for XAFS and XRF spectroscopic techniques (Harfouche *et al.*, 2018 ▸; El-Hasan *et al.*, 2021 ▸; Jamil *et al.*, 2021 ▸). In the XAFS experiment, the energy of the Zn *K*-edge (9659 eV) was calibrated using a Zn metal foil at the beamline. Similarly, a Mn metal foil was used for energy calibration at the Mn *K*-edge (6539 eV). Room-temperature XAFS data collection was accomplished over the Zn and Mn *K*-edges using transmission mode and fluorescence geometry, respectively. Fluorescence mode was used in the case of the Mn *K*-edge data collection in order to acquire a reasonable XAFS signal for a small concentration of Mn ions rather than using transmission mode. During the XAFS data collection, an energy step of 5 eV in the pre-edge part, 0.2 eV in the XANES portion, and a fixed *k*-step of 0.03 Å were employed. Five scans for each sample were registered to reinforce the signal-to-noise ratio. Firstly, for identifying the possible oxidation states of the Zn and Mn cations within the samples, we used XANES qualitative analysis and plotted the fingerprint XANES spectrum of the investigated sample with those of the collected element standard compounds. A Fourier transform (FT) was used to derive the χ(*R*) versus *R* spectra from the absorption spectra [using *ATHENA* software (Ravel & Newville, 2005 ▸)], generation of the theoretical EXAFS spectra starting from an assumed crystallographic structure, and finally fitting of the experimental data with the theoretical spectra using *ARTEMIS* software (Ravel & Newville, 2005 ▸). EXAFS data pre-analysis was performed using a set of standard procedures {pre-edge absorption removal, background reduction, normalization, EXAFS signal extraction, energy conversion into *k*, weighting scheme, and Fourier filtering or FT [χ(*R*) versus *R* spectra)] using the *ATHENA* program that is available within the *FEFF* (*DEMETER*) software package (Ravel & Newville, 2005 ▸; Rehr & Albers, 2000 ▸)}.

The threshold energy (*E*_0_) was recorded as a fraction of the edge step. FTs of the weighted EXAFS function were recorded using a Hanning window. EXAFS structural parameters (number of neighbors *N*, distance from the absorbing atom *R*, and Debye–Waller factor) for the first and second coordination shells around the absorbing ions were extracted from the curve-fitting procedure using experimental phases and amplitudes. The *ARTEMIS* software package (from *DEMETER*, Version 0.9.26) was used for modeling and structural fitting of the EXAFS oscillations. The best fits to the EXAFS signal were made in *R*-space in the interval 1.1–3.1 Å with Hanning windows within a 1–11 Å^−1^ *k*-interval, and all coordination shells up to a cut-off distance were included in the theoretical model. The fitting parameters included the amplitude reduction factor ( 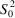 ) and correction to the difference in photoelectron energy (*E*_0_) between the experimental data and *FEFF* calculation, which were set similarly for all scattering paths. The interatomic distances (*R*) and disorder in the bond lengths or the mean-square relative displacements (MSRDs), also known as Debye–Waller factors (σ^2^), were well refined to extract the best-fit result. The theoretical EXAFS signals at the two metal absorption edges were calculated based on the ICDD crystallographic database for the ZnO NPs (Heiba *et al.*, 2015 ▸). In our fitting, 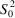 was determined first from the best fit of the first coordination shell and after that it was set fixed at the same value for higher coordination shells. Because 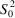 is due to intrinsic effects, it should be the same for every scattering path associated with a given absorbing atom (Heller *et al.*, 1950 ▸). Also, samples of the same composition should have the same value of 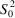 . For each sample to be ready for the XAFS experiment, a reasonable calculated amount of sample powder was homogeneously distributed into the PVP matrix. The sample/PVP composite was pressed into a pellet of diameter 13 mm and loaded into a sample holder for XAFS spectral data collection.

## 3. Results and discussion

### 3.1. XRD structural analysis

**Figure 1:**
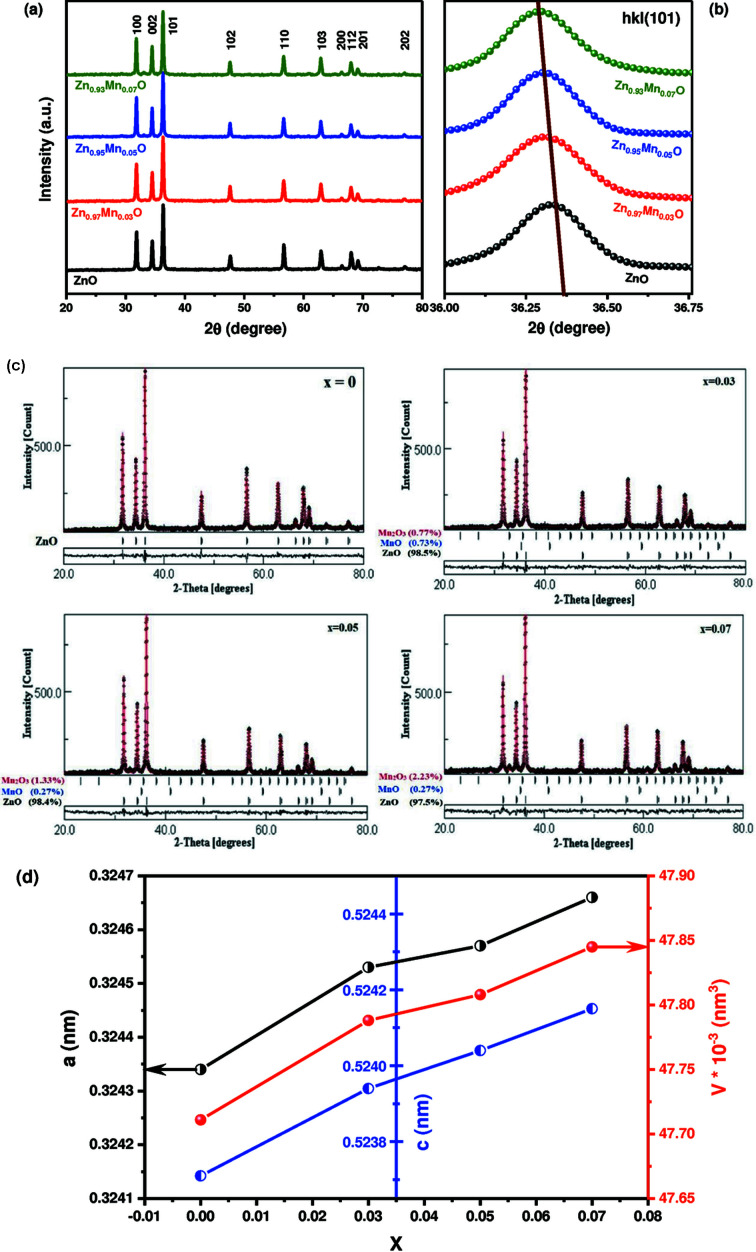
(*a*, *b*) XRD spectra, (*c*) Rietveld refinement, and (*d*) lattice parameters and unit-cell volume versus *x*.

The XRD analysis was carried out in the angular range 20° ≤ 2θ ≤ 80° to probe and confirm the formation of the desired Mn-doped ZnO NPs and determine the average crystal structural parameters; moreover, in order to check the phase purity of the formed Mn-doped ZnO NPs. The primary analysis of the XRD data is shown in Figs. 1 ▸(*a*) and 1(*b*). Fig. 1 ▸(*a*) shows the XRD peaks identifying the hexagonal structure of the Mn-doped ZnO with space group *P*63*mc*. The XRD patterns reveal the peak positions indexed for diffraction angles at 2θ = 31.79°, 34.47°, 36.29°, 47.55°, 56.61°, 62.88°, 66.39°, 67.95°, 69.10° and 76.98°, corresponding to reflections from the crystal planes of the identified hexagonal crystal structure. They match the standard ICDD #89-7102 card (Heller *et al.*, 1950 ▸). Fig. 1 ▸(*b*) shows the angular position shift of the most intensive diffraction peak for different Mn content (*x*). From a closer examination of the XRD patterns in Fig. 1 ▸(*b*), one can see that a higher Mn doping gives a smaller shift in angle. Gradual shifts toward smaller angles with an increase in the Mn doping concentration can be seen when looking at the position of the highly intense (101) peak. Rietveld refinement analysis of the XRD data using the *MAUD* program was performed to investigate the purity of the formed Mn-doped ZnO phase with the formula Zn_1–*x*_Mn*_x_* O, as depicted in Fig. 1 ▸(*c*) (Almoussawi *et al.*, 2020 ▸; Habanjar *et al.*, 2020 ▸). A small secondary phase of MnO and Mn_2_O_3_ was detected for *x* ≥ 0.03. As the Mn-dopant concentrations increased, the phase percentages of these secondary phases increased from 1.5% to 2.5%. The lattice parameters (*a*, *c* and *c*/*a*), lattice volume (*V*) and average crystallite size (*D*) of the hexagonal systems of Zn_1–*x*_Mn_*x*_O were extracted and are tabulated in Table 1 ▸ (Bilgili, 2019 ▸; Patterson, 1939 ▸). The average crystallite size *D* increases with the increment of the Mn content until *x* = 0.05, then decreases at *x* = 0.07. Further, Fig. 1 ▸(*d*) plots the variation of the lattice parameters *a*, *c* and unit-cell volume *V* as a function of Mn content in the ZnO host material. Lattice parameters *a* and *c* were slightly increased with increasing Mn content, resulting in a small increase in the unit-cell volume. The *c*/*a* parameter was calculated and found to be about 1.62, which is in close accord with the usual value of 1.633 for closed-packed hexagonal structures (Muniraja *et al.*, 2020 ▸). Equation (1) was used to calculate the atomic packing fraction (APF) (Mote *et al.*, 2013 ▸),

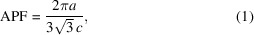

where *a* and *c* are lattice constants. The calculated APF values are summarized in Table 1 ▸. It is found that the APF increases as the Mn concentration increases, which may be attributed to a reduction in the sample voids. The APF of bulk hexagonal ZnO materials is around 74%, and the APF of the current samples is approximately 75%. This suggests that the APF in the nanocrystals is slightly higher than in the bulk materials, which may be due to the nanocrystalline samples’ size. The slightly increasing APF as the Mn content of Zn_1–*x*_Mn_*x*_O NPs increases indicates that Mn ions are substituted into the Zn sites of the ZnO structure in a homogeneous manner (Mote *et al.*, 2013 ▸).

The localization of the atoms and their displacements (*u*) in Zn_1–*x*_Mn_*x*_O structures and the lengths of the Zn—O bonds (*L*) were calculated using equations (2) and (3), respectively (Murtaza *et al.*, 2014 ▸),

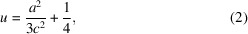




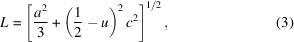

where *a* and *c* are lattice parameters and *u* is the positional parameter, which is a measure of the displacement of each atom relative to the next along the *c*-axis. With increasing Mn content, the bond length (Zn—O) increments slightly (Table 1 ▸), causing a small crystal deformation/imperfection in the ZnO crystal structure. A similar trend and a good agreement with the Zn—O bond length data have been observed in the literature (Bilgili, 2019 ▸; Senol *et al.*, 2020 ▸). The parameter *u* was computed and exhibited a constant value of 0.3779 for all samples (Table 1 ▸). The surface area to volume ratio (*S*/*V*) of the Zn_1–*x*_Mn_*x*_O NPs was determined using equation (4) (Mote *et al.*, 2013 ▸),

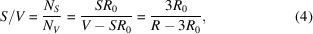

where *N*_S_ and *N*_V_ are the number of ZnO pairs on the surface and in the volume, respectively, *S* is the surface area, *V* is the unit-cell volume, *R* is the average particle radius, and *R*_0_ is the ZnO distance (0.018 Å). Values of the surface area to volume ratio were determined and are tabulated in Table 1 ▸. The *S*/*V* value decreasing with the increment of the Mn concentration could be related to the increase in average crystallite size of the Zn_1–*x*_Mn_*x*_O NPS. The calculated values of the *S*/*V* ratio are very small which means that the Zn_1–*x*_Mn_*x*_O NPs are spherical in shape. Similar results have been reported in the literature (Kazemi *et al.*, 2010 ▸).

### 3.2. XAFS data analysis and interpretation

#### 3.2.1. Zn *K*-edge XAFS spectral analysis

**Figure 2:**
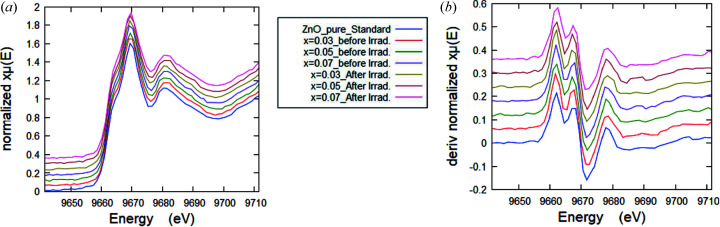
(*a*) Normalized XANES spectra of Mn-doped ZnO samples before and after irradiation, aligned vertically for clarification. (*b*) First derivative of the Zn *K*-edge XANES spectra with *y*-offset, compared with the first-derivative XANES spectrum of the ZnO standard compound.

Pre-absorption-edge (pre-peak) analysis and fitting, XANES spectra fingerprinting, along with those of the standard spectra, FT of the EXAFS signal, and fitting of the EXAFS oscillations were all performed. We observed a non-significant chemical shift of the edge position in correlation with the increment of the *x* value, giving a unique oxidation state for Zn^2+^. In more detail, the XANES spectra of the pristine and γ-irradiated ZnO doped with Mn samples at different Mn content (*x*) are aligned vertically as shown in Fig. 2 ▸(*a*); Fig. 2 ▸(*b*) displays the first derivative of the Zn *K*-edge XANES spectra of the samples, along with that of the ZnO standard compound. The extracted values of the spectral white-line position and the absorption energy, which was set as a fraction (half) of the normalized edge step of the Zn *K*-edge XANES spectra (Arman *et al.*, 2020 ▸), and also the oxidation state of the Zn ions, are shown in Table 2 ▸. It is clear that there is a non-significant chemical shift of the pre-edge and the main absorption edge positions in correlation with exposure to γ-ray irradiation and with the increment of the *x* value of the Mn content, giving a divalent oxidation state of Zn^2+^.

**Figure 3:**
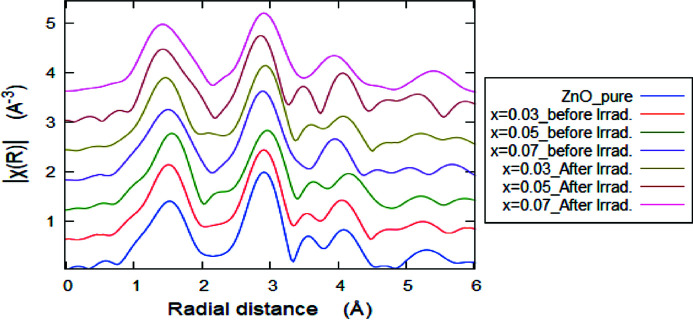
FTs corresponding to *k* ^2^-weighted EXAFS oscillations of Mn-doped ZnO samples at the Zn *K*-edge with *y*-offset for clarification.

**Figure 4:**
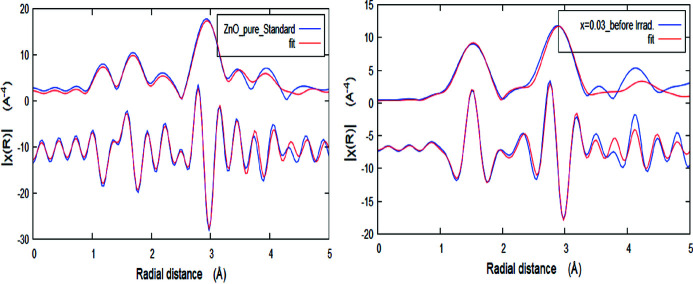
Examples of the EXAFS fit obtained for the FT magnitude and the imaginary part of the EXAFS signal at *x* = 0.0 and 0.03.

FT spectra corresponding to *k* ^2^-weighted EXAFS signals for the Mn-doped ZnO at the Zn *K*-edge are shown in Fig. 3 ▸. The input structural data file for the *ARTEMIS* program was built using ICDD #89-7102 of the ZnO standard (Jamil *et al.*, 2021 ▸; Ravel & Newville, 2005 ▸). Fig. 4 ▸ shows the best fit of the experimental χ(*R*) versus *R* spectra, using a Hanning window in the fitting range *R* = 1.0–3.5 Å. Examples of the EXAFS fit were obtained for the FT magnitude and the imaginary part of the EXAFS signal at *x* = 0.0 and 0.03. The best-fit EXAFS extracted fine structural parameters at the Zn *K*-edge are summarized in Table 3 ▸, where the bond distance (*R*), coordination number (*N*) and the mean square fluctuation in the bond distance (σ^2^) are used as fitting parameters. It is remarkable that the shapes of the FTs of the *k* ^2^χ(*k*)-weighted EXAFS signals for all samples are quite similar to that of the standard ZnO sample except at *x* = 0.07, either before or after irradiation. Generally, the FT amplitude reduced upon increasing the Mn content due to the reduction in the coordination number of the surrounding Zn atoms around the photoabsorber. Also, the FT amplitude reduced after γ-ray irradiation for all Mn-doped ZnO samples, which is linked to the reduction in the coordination number *N* around the Zn ions. This may arise from irradiation-induced structural disordering. The first coordination shell expressed by the first peak in the Zn *K*-edge FT-EXAFS signal (positioned around 1.97 Å) corresponds to the four oxygen ions surrounding the Zn cation [Zn–O1.1 (*N* = 4)]. The second FT peak is fitted with a single Zn–Zn/Mn shell as can be seen from Table 3 ▸. Therefore, for the Zn *K*-edge EXAFS data, there is a small change in the bond lengths with the increase in the Mn doping content (*x*) and after irradiation. It is observed that the mean-square relative displacement (σ^2^) increases from 0.001 Å^2^ to 0.014 Å^2^ for Zn—Mn bonds. This increase is in correlation with the compositional (Mn doping) and irradiation co-dependence local structure disorder, since σ^2^ is a measure of the disordering within a substance.

#### 3.2.2. Mn *K*-edge XAFs spectral analysis

**Figure 5:**
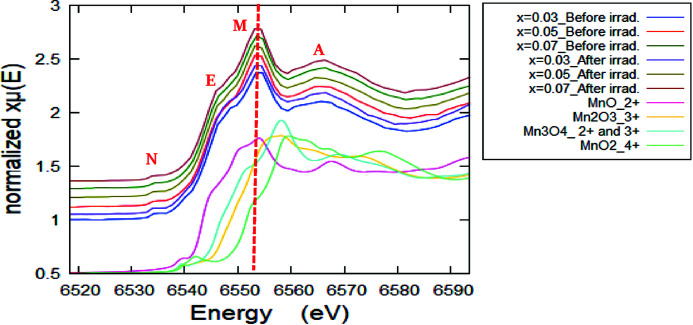
Normalized fingerprint XANES spectra of the pristine and γ-irradiated Mn-doped ZnO NPs for different Mn content (*x*) along with those of Mn-bearing reference compounds.

**Figure 6:**
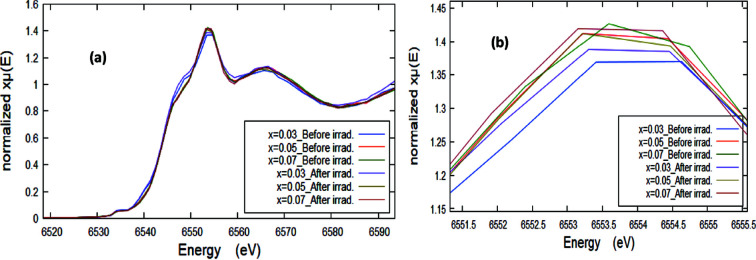
Normalized XANES spectra of samples before and after irradiation without *y*-offset for comparing the main absorption edge intensity at the Mn *K*-edge.

**Figure 7:**
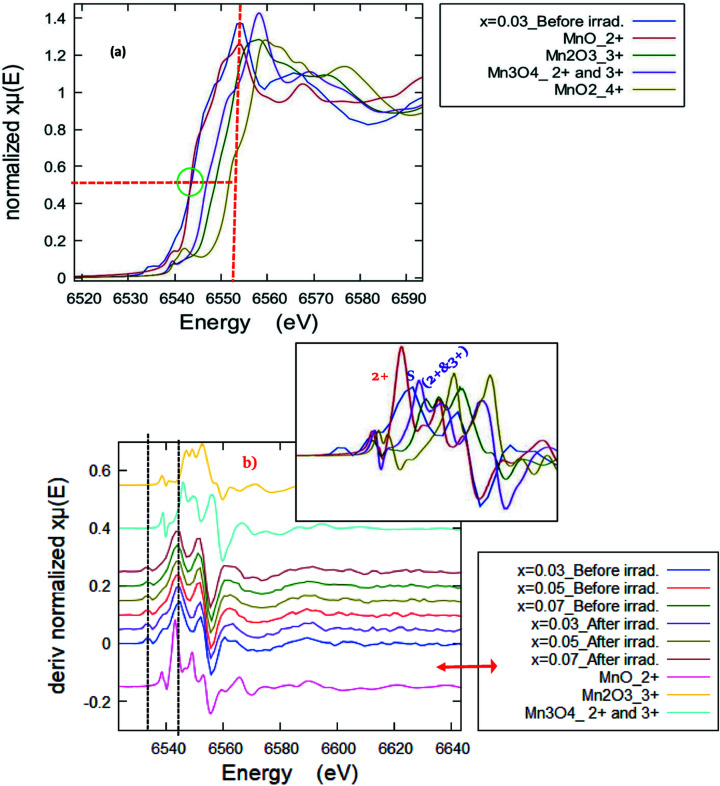
(*a*) Normalized fingerprint XANES spectra for *x* = 0.3 before irradiation along with the reference Mn compounds (with *y*-offset for clarification). (*b*) First derivative of Mn *K*-edge XANES spectra of Mn-doped ZnO samples before and after irradiation at different *x* along with those of reference Mn compounds. The inset is a zoom without offset.

**Figure 8:**
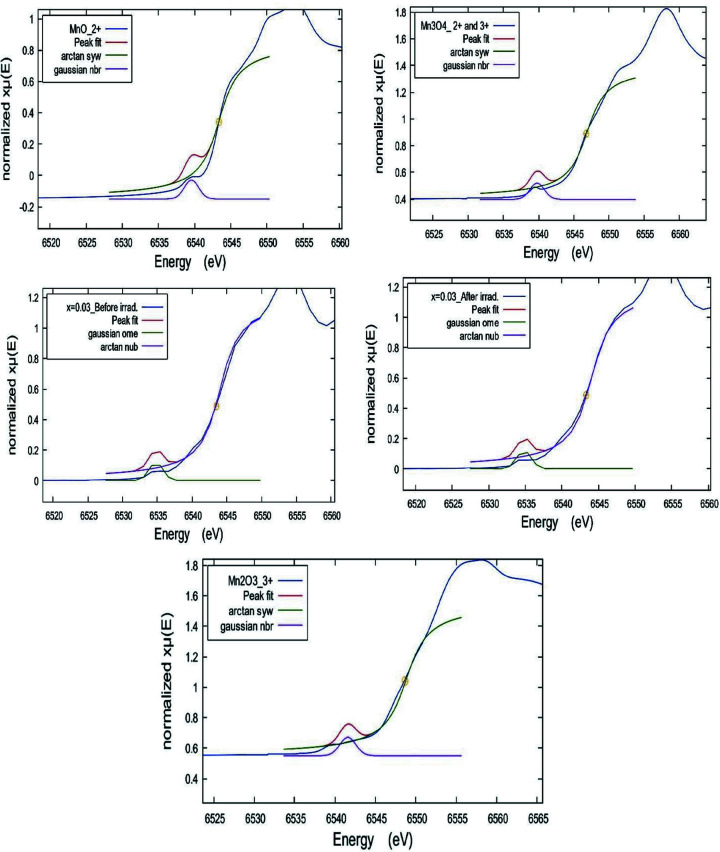
Pre-peak fit of the pristine and irradiated example sample at *x* = 0.03, along with those of Mn reference standards.

Fig. 5 ▸ shows normalized fingerprint XANES spectra (at the Mn *K*-edge) of the pristine and γ-ray irradiated Zn_1–*x*_Mn_*x*_O NPs for different Mn content (*x*) along with those of Mn-bearing reference compounds representing different oxidation states of Mn cations for comparison. In order to analyze the XANES spectra of unirradiated and irradiated Zn_1–*x*_Mn_*x*_O NPs at the Mn *K*-edge, the XANES spectra are plotted again in Fig. 6 ▸ without a *y*-offset for comparing the absorption peak amplitude. In fact, there is no obvious significant variation in the XANES spectral features after the Mn content change and also upon irradiation. On the other hand, a pre-edge peak generally appears at about 10–15 eV before the main absorption edge position (Morales-Pérez *et al.*, 2021 ▸). In the present case, the pre-edge peak of the Mn *K*-edge XANES spectra appears around 6534.8 eV with an intensity that does not alter much with increasing Mn content and after γ-ray irradiation, thus showing that the degree of hybridization and therefore the symmetry of the Mn cations does not show a significant variation upon irradiation or with changing Mn content (*x*). Generally, the XANES part is usually used for identifying the oxidation state of the photoabsorber (Imam *et al.*, 2021*c* ▸). The collected XANES fingerprint spectra of the samples were compared with those of the different Mn reference standards as shown in Fig. 6 ▸. For more clarification, only one XANES sample spectrum (*x* = 0.03 before irradiation) along with those of the Mn reference standards has been plotted in Fig. 7 ▸(*a*). It can be observed that the absorption edges of the Mn-doped ZnO samples are located between those of Mn^2+^ and (Mn^2+^ & Mn^3+^) bearing standard compounds, but very close to the Mn^2+^ absorption edge energy value. The values of the absorption edge were determined and are listed in Table 2 ▸. Further, in order to confirm the oxidation state(s) of the Mn ions in the samples, the first derivative of the XANES spectra [dμ(*E*)/d*E*] is necessary to provide a more accurate and distinguished absorption peak position of both the sample and the standards, compared with the original XANES collected spectra (Imam *et al.*, 2021*a* ▸). Consequently, Fig. 7 ▸(*b*) shows the first derivative [dμ(*E*)/d*E*] of the Mn *K*-edge XANES spectra of the ZnO NPs doped with Mn before and after irradiation at different *x* along with those of the reference Mn standards. The insert is a zoom without offset. The observed most intensive inflection point corresponding to the maximum of the white-line position of the example samples is very close to that of the Mn^2+^ standard, but in between those of Mn^3+^ and (Mn^2+^ & Mn^3+^), confirming the existence of trivalent Mn cations beside Mn^2+^. For more clarification, the oxidation state(s) of the Mn cations could be easily identified from the pre-edge fitting analysis that was performed using *ATHENA* software (Imam *et al.*, 2021*b* ▸). The pre-peak fit of the pristine and irradiated example sample at *x* = 0.03, along with that of the Mn reference standards, is shown in Fig. 8 ▸. In this pre-edge fitting procedure, the pre-edge peak is fitted to a Gaussian function. Herein, an arctan function was used first as a step function and then the pre-edge peak was fitted to a Gaussian function. The fitted centroid of the pre-edge for all investigated samples along with the different Mn standards are listed in Table 2 ▸. Not much difference in the centroid energy value of the pre-edge with the compositional change and irradiation effect is observed, suggesting the chemical coordination and structural symmetry remain invariant. On the other hand, the sample pre-edge centroid position is close to that of the Mn^2+^ standard, suggesting a divalent oxidation state of Mn of (2+).

**Figure 9:**
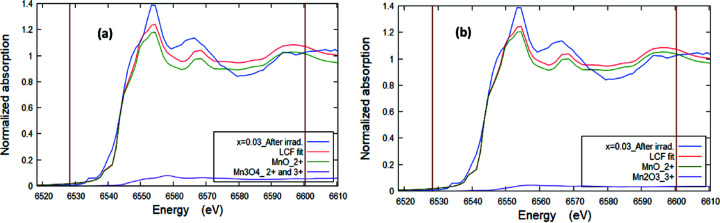
Linear combination fitting (LCF) of the XANES signal at the Mn *K*-edge with respect to the standard MnCl_2_ and Mn_3_O_4_ compounds (*a*), and to MnCl_2_ and Mn_2_O_3_ standards (*b*), respectively. The XANES signal in the range −20 eV to 57 eV of the irradiated sample at *x* = 0.03 is a representative example.

Further analysis was necessary for deciding whether or not there is an Mn^3+^ phase within the samples. Accordingly, linear combination fitting (LCF) analysis was performed in the range from −20 eV to 57 eV of the XANES spectra of the samples (Monged *et al.*, 2022 ▸). The refined LCF components were Mn^2+^ and (Mn^2+^ & M^3+^) reference standards once, and Mn^2+^ and Mn^3+^ once again as shown in Fig. 9 ▸, where the LCF was carried out with the aim of judging whether there is an impurity phase or not, and, if so, what the chemical formula is, and to determine the contribution percentage of the impurity phase in the collected XANES signal (Imam *et al.*, 2021*a* ▸). The results of the LCF analysis are summarized in Table 4 ▸. It was found that the best-fit LCF components were the Mn^2+^ and (Mn^2+^ & Mn^3+^) reference standards. Therefore, the potential impurity phase formula containing Mn^3+^ is Mn_3_O_4_, not Mn_2_O_3_. However, Table 4 ▸ shows the fitted percentage of each potential phase contributing to the collected XANES signal, and the percentage of Mn_3_O_4_ impurity phase reduces upon γ-ray irradiation for the same Mn content (*x*), along with a reduction of Mn^3+^ into Mn^2+^. The existence of Mn_3_O_4_ secondary phase means that there are zinc and oxygen vacancies affecting the magnetic properties, whereas the Mn_3_O_4_ percentage increases with increasing Mn content (*x*) from 0.03 to 0.05, both for the pristine and the γ-irradiated samples. Carefully examining the extracted information shown in Table 4 ▸ for the LCF analysis, one can conclude that the irradiated sample with *x* = 0.03 shows the lowest percentage amount of the impurity phase of Mn_3_O_4_ due to the reduction effect of γ-irradiation which transfers a portion of Mn^3+^ to Mn^2+^. Also, the most disordered samples which contain the highest percentage of Zn and O vacancies are the samples with *x* = 0.05 and 0.07, before irradiation. These findings support the structure parameters extracted from the Zn *K*-edge EXAFS analysis (Table 4 ▸).

Consequently, the potential formulas of the different samples based on the LCF results are shown in Table 4 ▸. The general sample formula is: 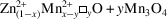 , where 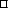 represents the zinc (Zn^2+^) vacancy due to the structural disorder which was proposed to saturate the vacant Zn sites that were not filled with Mn^2+^.

**Figure 10:**
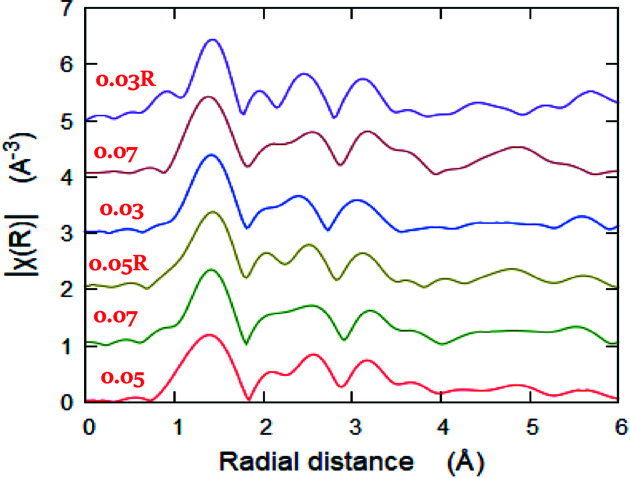
*k* ^2^-weighted EXAFS oscillations of Mn-doped ZnO samples at the Mn *K*-edge, shifted and arranged vertically according to their FT amplitudes.

The FTs of the *k* ^2^χ(*k*)-weighted EXAFS signals of Mn-doped ZnO at the Mn *K*-edge are shown in Fig. 10 ▸, shifted and arranged vertically according to their observed FT amplitudes. It is clear that the sample with *x* = 0.03R (R stands for after irradiation) shows the maximum FT amplitude, suggesting the highest coordination number of the surrounding atoms (Imam *et al.*, 2019 ▸). On the contrary, the samples with *x* = 0.05 and 0.07 show the lowest values of FT amplitude, suggesting the lowest coordination number of the surrounding atoms around the Mn absorbers. These results support those concluded from the LCF analysis of the Mn *K*-edge XANES spectra and the Zn *K*-edge EXAFS fitting. Also, there is a shift to lower values in the interatomic distances in the FTs of the samples at *x* = 0.05 and 0.07R confirming the above results that these samples are the most disordered samples. Therefore, the chemical coordination depends on the Mn concentration and the γ-irradiation effect.

From the XAFS data analysis, it is interesting to observe that there is a compositional and an irradiation co-dependence in the Mn-doped ZnO NPs. With increasing Mn content, the coordination number increases. Also, irradiation creates a significant structural change by producing structural imperfections such as vacancies and interstitials. The structural imperfections are detected from the XAFS, either from XANES LCF and/or EXAFS signal analysis and fitting. The electronic/local fine structure of the Zn and Mn ions is described by fitting the nearest and next-nearest coordination shells. Zn—O and Zn—Zn/Mn bond lengths are determined from the EXAFS fitting at the Zn *K*-edge. There is a dependence of the bond lengths on the Mn concentration (*x*) and upon γ-irradiation. In summary, Mn incorporation into the ZnO structure was confirmed from the XANES and EXAFS fitting. These results match well with those shown by Yadav *et al.* (2015 ▸).

### 3.3. Magnetic properties

**Figure 11:**
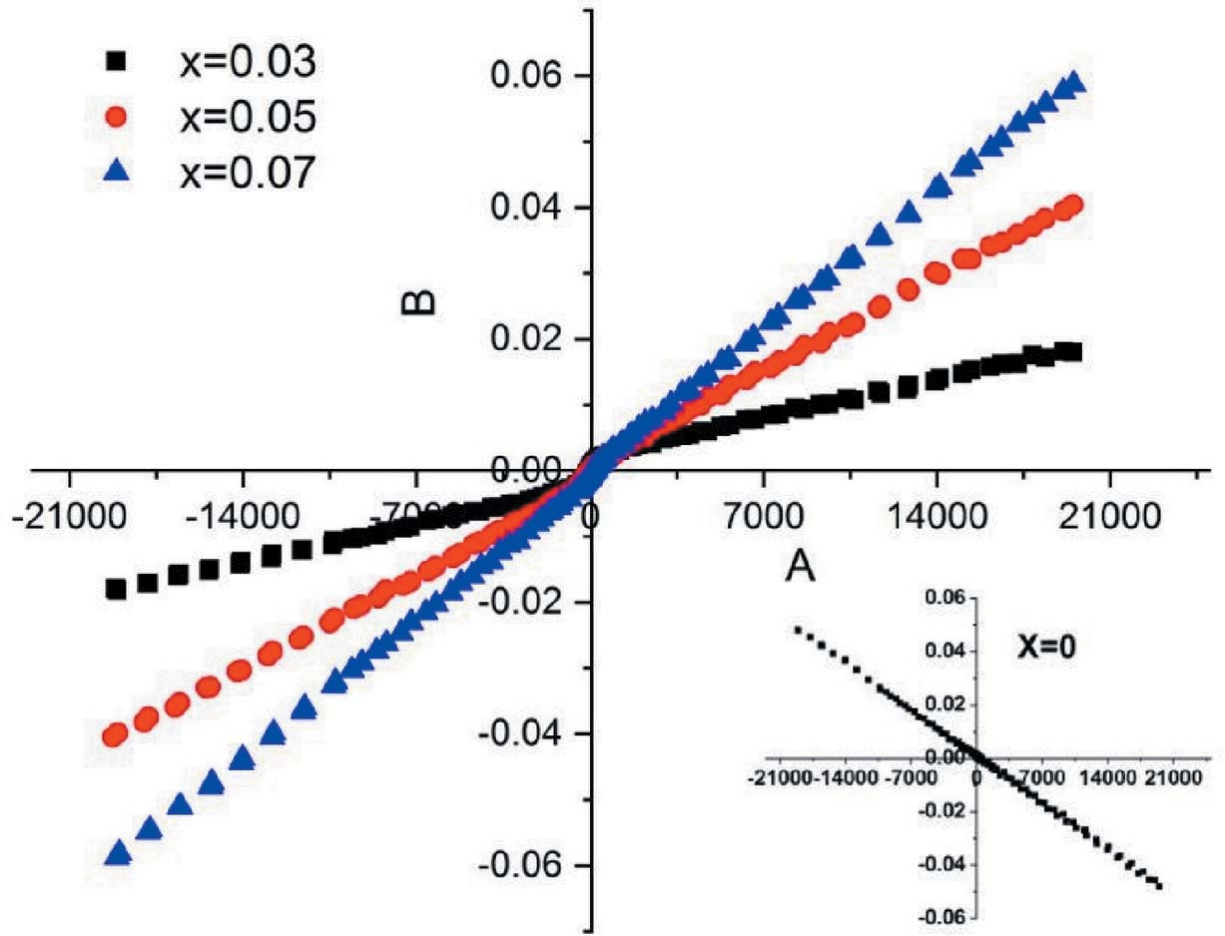
Room-temperature *M*–*H* loops of Mn-doped ZnO NPs (*x* = 0.0, 0.03, 0.05 and 0.07 doping).

The acquired magnetization curves of *M*–*H* loops at room temperature using the VSM technique for the Zn_1–*x*_Mn_*x*_O (*x* = 0.0, 0.03, 0.05 and 0.07) NPs are shown in Fig. 11 ▸. VSM analysis for undoped ZnO nanostructures shows a negative linear behavior at room temperature which indicates a diamagnetic behavior of ZnO nanostructures that has been reported by several research groups (Gandhi *et al.*, 2014 ▸; Zhou *et al.*, 2007 ▸; Azab *et al.*, 2018 ▸, 2019 ▸). The reason for this is that the paired electrons of the 2*p* orbital of oxygen and 3*d* orbital of zinc combine to form ZnO. No unpaired electrons are available to produce ferromagnetism. The behavior of the magnetization for doped samples (*x* = 0.03, 0.05 and 0.07) shows a weak ferromagnetic nature with unsaturated characteristics, where the magnetization at the maximum field (*M*_max_) increases with increasing Mn content. The coercivity values were 15.6, 64 and 1.1 Oe for *x* = 0.03, 0.05 and 0.07, respectively. It is still unclear what causes room-temperature ferromagnetism in the Mn-doped ZnO DMSs (Wei *et al.*, 2008 ▸). Many scientists thought that ferromagnetism at room temperature was due to the secondary phase (Kolesnik *et al.*, 2002 ▸; Li *et al.*, 2006 ▸; Kim *et al.*, 2004 ▸) that was discovered from XAFS analysis (Mn_3_O_4_). The EXAFS measurements revealed a relatively small amount of Mn_3_O_4_ as a secondary phase and showed the existence and increase of crystal disordering upon Mn doping. The previous reports illustrated that Mn_3_O_4_ has paramagnetic characteristics at room temperature which means that there is no contribution of Mn_3_O_4_ in the magnetic properties of Mn-doped ZnO under investigation (Guo *et al.*, 2000 ▸; Ichiyanagi *et al.*, 2006 ▸; Srinivasan & Seehra, 1983 ▸). Other researchers, on the other hand, believed that ferromagnetism resulted from the interaction of Mn^2+^ ions with crystal defects (Duan *et al.*, 2008 ▸; Kittilstved *et al.*, 2006 ▸; Babić-Stojić *et al.*, 2008 ▸; Coey *et al.*, 2005 ▸); therefore, the magnetism is not originating directly from the secondary phase itself but indirectly from the induced crystal defects in the Mn-doped ZnO nanocrystal. XANES and EXAFS analysis showed that Mn^2+^ forms the majority of Mn ions in all samples, as illustrated in Table 4 ▸. In DMS, the exchange interactions between the defects and the surrounding Mn^2+^ ions generate a bound magnetic polaron (BMP), which can overlap and cause long-range Mn^2+^–Mn^2+^ ferromagnetic coupling. Strong ferromagnetism can only be produced when the concentration of Mn^2+^ ions and defects is within an appropriate range (Schmidt-Mende & MacManus-Driscoll, 2007 ▸; Khorsand Zak *et al.*, 2012 ▸). As the Mn^2+^ concentration increases, XANES and EXAFS showed that the concentration of the defects has also increased, and more BMPs will be formed and overlap, so a higher *M*_max_ is observed in Mn-doped samples. Meanwhile, the increase of Mn content may lead to Mn clusters in the ZnO matrix. Many authors (Harsono *et al.*, 2017 ▸; Luo *et al.*, 2014 ▸; Subramanian *et al.*, 2011 ▸; Hao *et al.*, 2012 ▸) have also reported the same trend for our samples.

## 4. Conclusion

This paper describes XAFS data that have been collected at the XAFS/XRF beamline at SESAME. Two different kinds of radiation (γ-rays and synchrotron radiation) were used for irradiation (tailoring) and then structural characterization, respectively. Mn-doped ZnO NPs were tailored by inducing structure disorder with γ-rays (200 K Gy) for more magnetic functionality. XAFS at SESAME provided detailed fine (local/electronic) structural information of the pristine and γ-ray irradiated Mn-doped ZnO NPs. The magnetic behavior of Mn-doped ZnO NPs was explained in light of the XRD and XAFS results. XRD demonstrated the successful preparation of hexagonal Mn-doped ZnO NPs with a crystallite size in the range 33–41 nm. XRD data analysis predicted the existence of a small amount of the secondary phase of Mn_3_O_4_. XAFS (XANES and EXAFS) fitting confirmed the existence of Mn_3_O_4_ as a secondary phase. There was a strong harmony among the XRD, XANES, EXAFS and magnetization behavior of Mn-doped ZnO NPs. The compositional change upon Mn doping, along with (γ-irradiation) induced lattice defects (Zn and O vacancies), improved the magnetic character of the ZnO NPs. The composition at 0.05 Mn doping showed the most enhanced magnetization due to the induced maximum lattice imperfections.

## Figures and Tables

**Table 1:** Lattice parameters (*a*, *c*), average NP size (*D*), unit-cell volume (*V*), atomic packing fraction (APF), positional parameter (*u*), Zn—O bond length (*L*) and dislocation density (δ) of Zn_1–*x*_Mn_*x*_O NPs

Sample	ZnO	Zn_0.97_Mn_0.03_O	Zn_0.95_Mn_0.05_O	Zn_0.93_Mn_0.07_O
*a* = *b* (nm)	0.3243	0.3245	0.3246	0.3240
*c* (nm)	0.5237	0.5239	0.5240	0.5242
*c*/*a*	1.615	1.614	1.615	1.614
*D* (nm)	32.81	35.57	41.21	39.58
*V* (×10^−3^ nm^3^)	47.711	47.788	47.808	47.845
APF	0.74887	0.74898	0.74894	0.749
*u*	0.3779	0.3779	0.3779	0.3779
*L* (nm)	0.1979	0.1980	0.1980	0.1981
δ (×10^−3^ nm^−2^)	0.92889	0.79033	0.58871	0.6384
*S*/*V*	0.02459	0.02458	0.02457	0.02456

**Table 2:** Fitted pre-edge peak fitting of the different samples along with reference model compounds bearing Mn with different oxidation states, spectral white line position, absorption energies set as a fraction (half) of the edge step, oxidation states of the Zn and Mn cations in the reference compounds, and samples at both Zn and Mn *K*-edges

Group (*x*)	Fitted pre-edge centroid *E* (eV)		White-line position (eV) ±0.02	*E*_0_ (eV), selected at the half-height of the edge step ±0.03	Oxidation state
Zn_ *K*-edge (9659 eV) – tabulated value					
*x* = 0.03, before irradiation	–		9669.52	9661.62	Zn^2+^
*x* = 0.05, before irradiation	–		9669.49	9661.62	Zn^2+^
*x* = 0.07, before irradiation	–		9669.52	9661.69	Zn^2+^
*x* = 0.03, after irradiation	–		9669.48	9661.61	Zn^2+^
*x* = 0.05, after irradiation	–		9669.53		Zn^2+^
*x* = 0.07, after irradiation	–		9669.53		Zn^2+^
ZnO pure standard	–		9669.68	9661.58	Zn^2+^

Mn *K*-edge (6539 eV) – tabulated value					
*x* = 0.03, before irradiation	6534.80 ± 1.76		6554.03	6543.52	Mn^2+^
*x* = 0.05, before irradiation	6534.80 ± 1.76		6553.75	6543.51	Mn^2+^
*x* = 0.07, before irradiation	6534.80 ± 2.66		6553.90	6543.50	Mn^2+^
*x* = 0.03, after irradiation	6534.80 ± 2.64		6553.85	6543.33	Mn^2+^
*x* = 0.05, after irradiation	6534.80 ± 1.93		6553.69	6543.50	Mn^2+^
*x* = 0.07, after irradiation	6534.80 ± 1.97		6553.76	6543.45	Mn^2+^
Mn-foil	–		6555.17	6543.47†	Mn^0^
MnO	6539.56 ± 0.79		6553.95	6543.37	Mn^2+^
Mn_2_O_3_	6541.59 ± 1.01		6558.13	6548.66	Mn^3+^
Mn_3_O_4_	6539.79 ± 0.60		6558.17	6546.80	Mn^2+^ and Mn^3+^
MnO_2_	6542.06 ± 0.01		6559.49	6551.62	Mn^4+^

† The inflection point of the pre-edge peak is 6538 eV.

**Table 3:** The best-fit model parameters of the EXAFS signals showing the compositional dependence of the Mn dopant at different values of *x* with respect to the pure ZnO for pristine and irradiated samples

Path	*N* (atoms)	σ^2^ (Å^2^) ± 0.002	*R* (Å) ± 0.012
Pure ZnO, 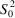 = 1.0, Δ*E*_0_ = 6 ± 0.02 eV, *R*-factor = 0.02			
Zn–O1.1	4	0.007	1.981
Zn–Zn0.1	12	0.013	3.23
Zn–O1.4	9	0.007	3.80

ZnO:Mn (0.03) before irradiation, 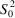 = 0.9, Δ*E*_0_ = 5.46 ± 0.02 eV, *R*-factor = 0.02, *K* = 2–10, *r* = 1–3.5, Hanning window			
Zn–O1.1	4.00	0.004	1.975
Zn–Zn0.1	1.00	0.014	3.284
Zn–O1.4	9.00	0.004	3.794
Zn–Mn0.1	1.00	0.001	3.284

ZnO:Mn (0.03) after irradiation, 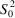 = 0.9, Δ*E*_0_ = 5.46 ± 0.02 eV, *R*-factor = 0.03, *K* = 2–10, *r* = 1–3.5, Hanning window			
Zn–O1.1	4.00	0.005	1.972
Zn–Zn0.1	1.00	0.025	3.313
Zn–O1.4	9.00	0.005	3.788
Zn–Mn0.1	1.00	0.002	3.313

ZnO:Mn (0.05) before irradiation, 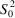 = 0.9, Δ*E*_0_ = 5.46 ± 0.02 eV, *R*-factor = 0.02, *K* = 2.1–12, *r* = 2.1–3.5, Hanning window			
Zn–O1.1	4.00	0.004	1.974
Zn–Zn0.1	1.00	0.023	3.317
Zn–O1.4	9.00	0.004	3.793
Zn–Mn0.1	1.00	0.004	3.317

ZnO:Mn (0.05) after irradiation, 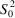 = 0.9, Δ*E*_0_ = 5.46 ± 0.02 eV, *R*-factor = 0.02, *K* = 2.1–12, *r* = 2.1–3.5, Hanning window			
Zn–O1.1	4.00	0.004	1.978
Zn–Zn0.1	1.00	0.015	3.294
Zn–O1.4	9.00	0.004	3.841
Zn–Mn0.1	1.00	0.005	3.294

ZnO:Mn (0.07) before irradiation, 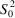 = 0.9, Δ*E*_0_ = 5.46 ± 0.02 eV, *R*-factor = 0.03, *K* = 2–10, *r* = 1–3.5, Hanning window			
Zn–O1.1	4.00	0.005	1.975
Zn–Zn0.1	1.00	0.014	3.300
Zn–O1.4	9.00	0.005	3.796
Zn–Mn0.1	1.00	0.0014	3.300

ZnO:Mn (0.07) after irradiation, 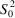 = 0.9, Δ*E*_0_ = 5.46 ± 0.02 eV, *R*-factor = 0.03, *K* = 2–10, *r* = 1–3.5, Hanning window			
Zn–O1.1	4.00	0.007	1.975
Zn–Zn0.1	1.00	0.014	3.295
Zn–O1.4	9.00	0.007	3.796
Zn–Mn0.1	1.00	0.014	3.295

**Table 4:** Contribution of Mn^2+^ and Mn^3+^ in the XANES signal collected at the Mn *K*-edge extracted from LCF analysis, and the corresponding sample formula

Group (*x*)	Mn^2+^ (%)	(Mn^2+^ & Mn^3+^) (%)	Mn^2+^ %	Mn^3+^ (%)	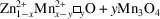 †
0.03	89.4 ± 5.0	10.6 ± 0.8	91.7 ± 4.3	8.3 ± 1.4	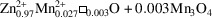
0.05	86.4 ± 5.0	13.6 ± 1.1	88.4 ± 4.2	11.6 ± 1.9	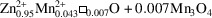
0.07	86.6 ± 5.0	13.4 ± 1.1	88.4 ± 4.3	11.6 ± 1.9	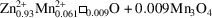
0.03R‡	94.4 ± 5.5	5.6 ± 0.4	96.6 ± 4.7	3.4 ± 0.5	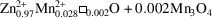
0.05R‡	87.5 (5.7)	12.5 ± 0.9	89.6 ± 4.3	10.4 ± 1.7	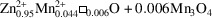
0.07R‡	90.9 ± 5.2	9.1 ± 0.7	91.8 ± 4.4	8.2 ± 1.4	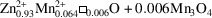

† The 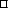 symbol represents the Zn vacancies equals to the amount % of Mn_3_O_4_ impurity phase.

‡ *R* is the γ-irradiated sample.
